# Supplementary material for: p1/s1, a 3’-nucleotidase/nuclease, allows Leishmania major to circumvent host innate immune response mechanisms
Source: PLoS Pathog. 2026 May 20;22(5):e1014197. doi: 10.1371/journal.ppat.1014197 (PMC13232948; doi:10.1371/journal.ppat.1014197)
Supplement: S1 Appendix — Phosphatidylserine exposure as cell death marker determined in flow cytometry by AnnexinV positivity (AnnV+) of promastigotes treated with 25 µM miltefosine or 25 µM staurosporine for the indicated time, compared to untreated promastigotes derived from the logPh or statPh. n = 3–6. Fig B – Effect of extracellular adenosine generated from 3’-AMP hydrolysis on hMDM infection. A, B- A2a and A2b positivity, respectively, of hMDM with different differentiations and stimulations, as specified. The surface markers were stained with respective specific and labeled antibodies for quantification in flow cytometry. Differentiation stimuli (50 ng/ml or 30 ng/ml GM-CSF) were applied for 5–7 days, followed by 24 h of activation with 50 ng/ml IFNγ or 100 ng/ml LPS, if applicable. n = 3–6 donors in 2–3 independent experiments. C- Absolute values to Fig 3A. hMDM (GM-CSF + IFNγ) were infected with MOI = 10 statPh L. majorDsRed promastigotes for 3 hours. During infection, adenosine (Ado) or 3’-AMP were maintained in the indicated concentrations. Soluble TNFα secreted from macrophages was determined in supernatant collected 3 hours post infection using ELISA. n = 7 donors in 4 independent experiments. D- Absolute values to Fig 3B. Relative parasite burden measured as DsRed MFI of infected hMDM (GM-CSF + IFNγ) normalized to the infected control without additives. Macrophages were infected for 3 hours with a MOI = 20 of statPh L. majorDsRed in the presence of adenosine or 3‘-AMP before autologous PBLs were added to the infection and incubated for 4 days. n=9-10 donors in 4 independent experiments. E- Absolute values to Fig 3C. hMDM (GM-CSF+IFNγ) in co-culture with autologous PBLs were infected with L. majorDsRed. Percentage of proliferated PBLs was determined as viable CellTracelow lymphocytes 4 days pi. n=9-10 donors in 4 independent experiments. Fig C – L. major p1/s1 null mutant endonuclease activity. Uncropped gel image to Fig 5B. Endonuclease activity measured by degradation of doub [file ppat.1014197.s001.docx]

Supplemental information for

**p1/s1, a 3’-nucleotidase/nuclease, allows *Leishmania major* to circumvent host innate immune response mechanisms**

Stella M. Schmelzle^1^, Michaela Bergmann^1^, Bianca Walber^1^, Jamal Shamsara^2,3^, Tanja Ziesmann^4, 5^, Ute Distler^4,5^, Csaba Miskey^6^, Liam Childs^7^, Peter Kolb^2,3^, Stefan Tenzer^4,5^, Katrin Bagola^1^, Ger van Zandbergen^1,4,5*^

1 Division of Immunology, Paul-Ehrlich-Institut, Langen, Germany

2 Institute for Pharmaceutical Chemistry, Philipps-Universität Marburg, Marburg, Germany

3 Core Facility "Chemoinformatics & Molecular AI", Philipps-Universität Marburg, Marburg, Germany

4 Institute for Immunology, University Medical Center, Johannes Gutenberg-University Mainz, Mainz, Germany

5 Research Center for Immunotherapy (FZI), University Medical Center, Johannes Gutenberg-University Mainz, Mainz, Germany

6 Di­vi­sion Haema­tol­o­gy, Cell and Gene Ther­a­py, Genomics Core Facility, Paul-Ehrlich-Institut, Langen, Germany

7 Research Group Host-Pathogen Interactions, Paul-Ehrlich-Institut, Langen, Germany

* [ger.vanzandbergen@pei.de](mailto:ger.vanzandbergen@pei.de)

**This file includes:**

S1 Appendix Fig A-F

S1 Appendix Table A-M

S1 Appendix Supplemental Methods

**Fig A**

**

**

**Fig A – Cell-death induced in *L. major* promastigotes**

Phosphatidylserine exposure as cell death marker determined in flow cytometry by AnnexinV positivity (AnnV+) of promastigotes treated with 25 µM miltefosine or 25 µM staurosporine for the indicated time, compared to untreated promastigotes derived from the logPh or statPh. n=3-6.

**Fig B**


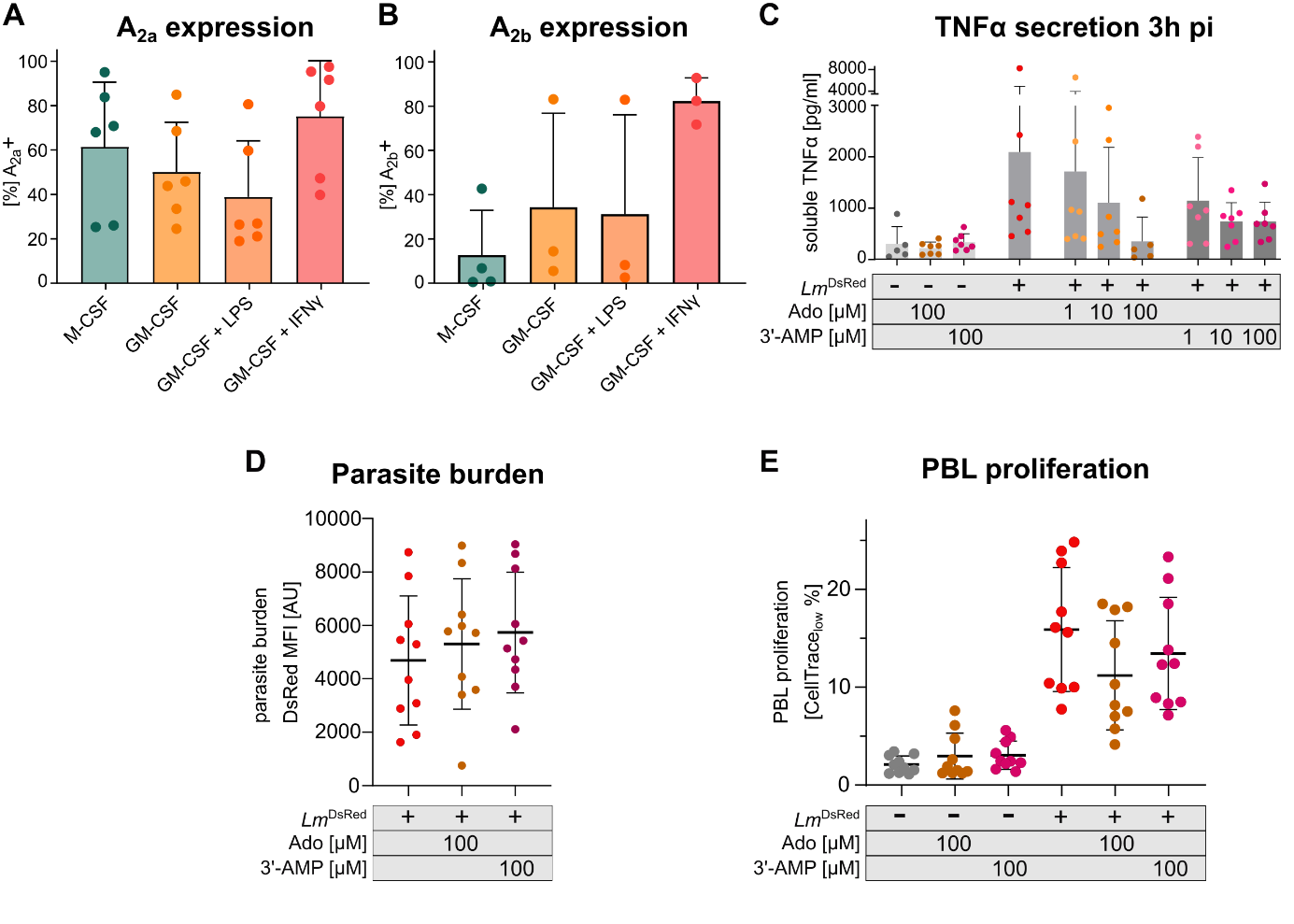


**Fig B – Effect of extracellular adenosine generated from 3’-AMP hydrolysis on hMDM infection**

**A, B**- A_2a_ and A_2b_ positivity, respectively, of hMDM with different differentiations and stimulations, as specified. The surface markers were stained with respective specific and labelled antibodies for quantification in flow cytometry. Differentiation stimuli (50 ng/ml or 30 ng/ml GM-CSF) were applied for 5-7 days, followed by 24 h of activation with 50 ng/ml IFNγ or 100 ng/ml LPS, if applicable. n=3-6 donors in 2-3 independent experiments.

**C**- Absolute values to Fig 3A. hMDM (GM-CSF+IFNγ) were infected with MOI=10 statPh *L. major*^DsRed^ promastigotes for 3 hours. During infection, adenosine (Ado) or 3’-AMP were maintained in the indicated concentrations. Soluble TNFα secreted from macrophages was determined in supernatant collected 3 hours post infection using ELISA. n=5-7 donors in 4 independent experiments.

**D**- Absolute values to Fig 3B. Relative parasite burden measured as DsRed MFI of infected hMDM (GM-CSF+IFNγ) normalized to the infected control without additives. Macrophages were infected for 3 hours with a MOI=20 of statPh *L. major*^DsRed^ in the presence of adenosine or 3‘-AMP before autologous PBLs were added to the infection and incubated for 4 days. n=9-10 donors in 4 independent experiments.

**E**- Absolute values to Fig 3C. hMDM (GM-CSF+IFNγ) in co-culture with autologous PBLs were infected with *L. major*^DsRed^. Percentage of proliferated PBLs was determined as viable CellTrace_low_ lymphocytes 4 days pi. n=9-10 donors in 4 independent experiments.

**Fig C**





**Fig C – *L. major* *p1/s1* null mutant endonuclease activity**

Uncropped gel image to Fig 5B.

Endonuclease activity measured by degradation of double-stranded M13 phage genome on 2x10^6^ total *L. major* statPh promastigotes (*L. major*^Cas9/T7/diCre/3‘flox^ *L. major^Δ^*^p1/s1^ clones E10 & F4 and *L. major*^p1/s1 addback^). Pellet and supernatant (SN) were separated and circular, double-stranded M13 DNA added for 60 min. Remaining intact substrate was assessed on a 0.8% TAE-agarose gel, semi-quantified using ImageJ and normalized to intact substrate not incubated with parasites (dsM13). Representative, uncropped gel shown. Also included are controls containing only parasites (*Lm)* and dsM13 incubated with DNase.

**Fig D**

**

**

**Fig D – Comparative proteomics of *L. major* *p1/s1* null mutants to parental strain**

**A**- Differentially detected proteins and intersections between parental strain *L. major*^Cas9/T7/diCre/3‘flox^ and *L. major^Δ^*^p1/s1^ clones E10 & F4 in a quantitative proteomics analysis.

**B**- Gene ontology enrichment of molecular functions for proteins with increased abundance in *Δp1/s1* iKO E10 compared to the parental strain.

**C**- Gene ontology enrichment of molecular functions for proteins with increased abundance in *Δp1/s1* iKO F4 compared to the parental strain.

Analysis based on 7 biological replicates and 3 technical replicates each.

**Fig E**


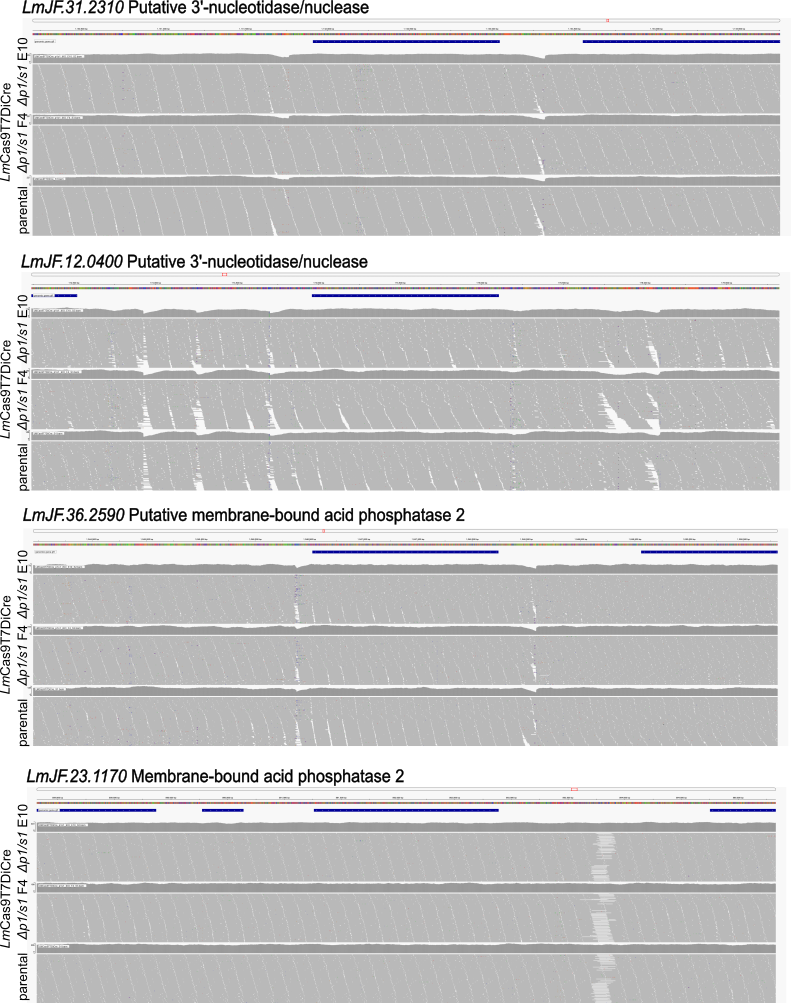


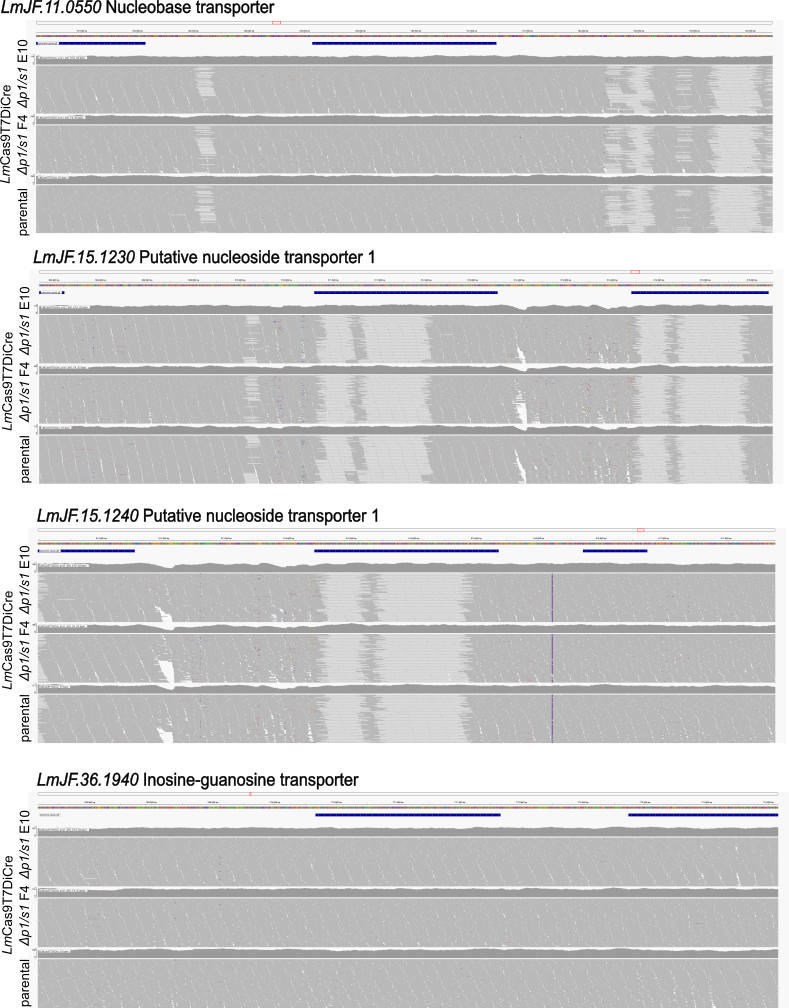


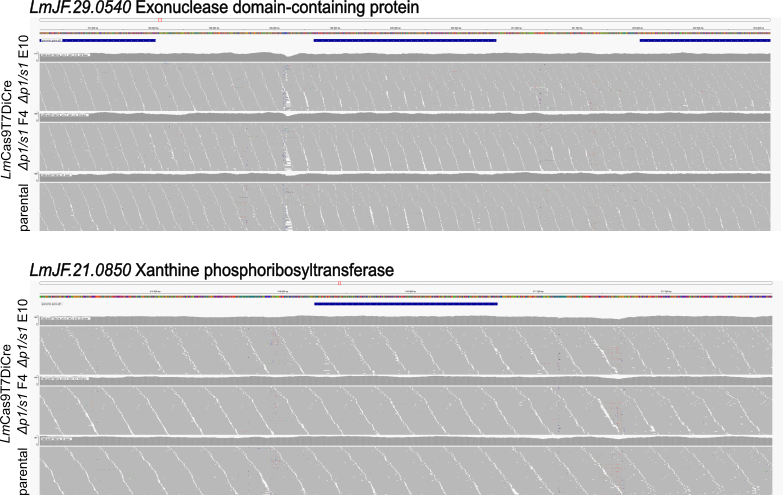


**Fig E – Mapped WGS reads of *L. major*^Δp1/s1^ and a parental strain at loci of interest**

Mapped whole genome sequencing (WGS) reads of a parental strain and *p1/s1* iKOs (*L. major*^Cas9/T7/diCre/3‘flox^ *L. major^Δ^*^p1/s1^ clones E10 & F4) at loci of interest as detailed in the figure. Loci of interest were identified by elevated protein levels in a comparative proteomics screen (Table 3)

**Fig F**





**Fig F – *L. major* *p1/s1* null mutants induce comparable PBL proliferation but fail to suppress proliferation with 3’-AMP as the parental strain.**

**A-** Proportion of metacyclic promastigotes within the infectious stationary phase (day 8, day9) of 2x10^8^ parental or p1/s1 iKO parasites as determined by peanut lectin purification.

**B-** Absolute values to Fig 8D. Macrophages were infected for 3 hours with MOI=20 of statPh promastigotes (*L. major*^Cas9/T7/diCre/3‘flox^, *L. major^Δ^*^p1/s1^ clones E10 & F4) in the presence of adenosine or 3‘-AMP before autologous CD14^-^ peripheral blood lymphocytes (PBLs) were added and incubated for 4 days. Percentage of proliferated PBLs was determined as CellTrace_low_. n=6 donors in 3 independent experiments.

**Table A: Data to Fig 3A - Soluble TNFα , normalized to infected, untreated**

| blood donor | un-infected | uninfected + 100µM Ado | uninfected + 100µM 3'AMP | L. major DsRed | L. major DsRed + 1µM Ado | L. major DsRed + 10µM Ado | L. major DsRed + 100µM Ado | L. major DsRed + 1µM 3'AMP | L. major DsRed + 10µM 3'AMP | L. major DsRed + 100µM 3'AMP |
| --- | --- | --- | --- | --- | --- | --- | --- | --- | --- | --- |
| d 319 | 0.37 | 0.12 | 0.26 | 1 | 0.95 | 0.96 | 0.49 | 0.91 | 0.56 | 0.61 |
| d 583 | 0.02 | 0.01 | 0.05 | 1 | 0.8 | 0.36 |  | 0.29 | 0.11 | 0.08 |
| d 944 | 0.22 | 0.57 | 0.56 | 1 | 0.9 | 0.54 | 0.09 | 0.68 | 0.76 | 0.75 |
| d 214 |  | 0.17 | 0.34 | 1 | 0.84 | 0.63 | 0.53 | 0.57 | 0.46 | 0.73 |
| d 865 | 0.05 | 0.11 | 0.17 | 1 | 0.88 | 0.47 | 0.19 | 0.9 | 0.8 | 0.66 |
| d 299 | 0.25 | 0.37 | 0.34 | 1 | 0.87 | 0.74 |  | 0.93 | 0.72 | 0.81 |
| d 763 |  | 0.33 | 0.35 | 1 | 0.49 | 0.67 | 0.08 | 1.01 | 0.82 | 0.85 |
| Mean | 0.18 | 0.24 | 0.30 | 1.00 | 0.82 | 0.62 | 0.28 | 0.76 | 0.60 | 0.64 |
| Std.  Dev. | 0.15 | 0.19 | 0.16 | 0.00 | 0.15 | 0.20 | 0.22 | 0.26 | 0.25 | 0.26 |

**Table B: Raw data to Fig BC in S1 Appendix - Soluble TNFα [pg/ml]**

| blood donor | un-infected | uninfected + 100µM Ado | uninfected + 100µM 3'AMP | L. major DsRed | L. major DsRed + 1µM Ado | L. major DsRed + 10µM Ado | L. major DsRed + 100µM Ado | L. major DsRed + 1µM 3'AMP | L. major DsRed + 10µM 3'AMP | L. major DsRed + 100µM 3'AMP |
| --- | --- | --- | --- | --- | --- | --- | --- | --- | --- | --- |
| d 319 | 885.64 | 299.95 | 630.49 | 2423.41 | 2296.3 | 2325.66 | 1181.58 | 2196.62 | 1348.14 | 1472.77 |
| d 583 | 174.34 | 103.22 | 451.39 | 8239.22 | 6552.56 | 2952.25 |  | 2389.11 | 903.13 | 646.51 |
| d 944 | 99.51 | 257.22 | 254.02 | 452.31 | 407.59 | 245.88 | 38.58 | 305.8 | 343.31 | 339.13 |
| d 214 |  | 93.72 | 184.12 | 536.31 | 452.85 | 338.44 | 282.78 | 306.53 | 245.75 | 390.83 |
| d 865 | 55.62 | 111.62 | 174.76 | 1055.41 | 929.58 | 493.64 | 195.85 | 954.18 | 846.54 | 697.26 |
| d 299 | 281.96 | 410.03 | 377.45 | 1116.98 | 966.29 | 830.71 | <15,6 | 1037.59 | 803.1 | 907.28 |
| d 763 | <15,6 | 266.05 | 287.3 | 810.73 | 399.68 | 539.68 | 66.86 | 822.83 | 663.03 | 692.87 |
| Mean | 249.5 | 220.3 | 337.1 | 2091 | 1715 | 1104 | 294.3 | 1145 | 736.1 | 735.2 |
| Std.  Dev. | 326.7 | 120.7 | 163.3 | 2789 | 2234 | 1080 | 447.4 | 837.9 | 369.5 | 378.6 |

**Table C: Data to Fig 3B – Parasite burden, normalized to untreated**

| blood donor | L. major DsRed | L. major DsRed + Ado | L. major DsRed + 3'AMP |
| --- | --- | --- | --- |
| d 839 | 1 | 1.03 | 1.03 |
| d 252 | 1 | 2 | 2.1 |
| d 269 | 1 | 1.05 | 0.87 |
| d 657 | 1 | 1.1 | 1.41 |
| d 018 | 1 | 0.76 | 1.04 |
| d 398 | 1 | 1.38 | 1.43 |
| d 765 | 1 | 1.21 | 0.97 |
| d 567 | 1 | 1.03 | 0.93 |
| d 731 | 1 | 2.19 | 1.3 |
| Mean | 1 | 1.306 | 1.231 |
| Std. Dev. | 0 | 0.4791 | 0.3869 |

**Table D: Raw data to Fig 3B and Fig BD in S1 Appendix – Parasite burden (DsRed MFI) [AU]**

| blood donor | L. major DsRed | L. major DsRed + Ado | L. major DsRed + 3'AMP |
| --- | --- | --- | --- |
| d 839 | 8737 | 8990 | 9040 |
| d 252 | 2889 | 5779 | 6055 |
| d 269 | 5453 | 5720 | 4728 |
| d 657 | 3090 | 3401 | 4343 |
| d 018 | 7849 | 5983 | 8132 |
| d 398 | 6053 | 8339 | 8678 |
| d 765 | 5297 | 6408 | 5140 |
| d 567 | 3964 | 4076 | 3704 |
| d 731 | 1633 | 3584 | 2116 |
| Mean | 4996 | 5809 | 5771 |
| Std. Dev. | 2345 | 1958 | 2397 |

**Table E: Data to Fig 3C – PBL proliferation, normalized to untreated**

| blood donor | L.major DsRed | L.major DsRed + Ado | L.major DsRed + 3'AMP |
| --- | --- | --- | --- |
| d 839 | 1 | 0.66 | 0.788 |
| d 915 | 1 | 0.324 | 0.701 |
| d 252 | 1 | 0.752 | 0.849 |
| d 269 | 1 | 0.746 | 0.94 |
| d 657 | 1 | 0.712 | 0.841 |
| d 018 | 1 | 0.901 | 0.857 |
| d 398 | 1 | 0.789 | 0.93 |
| d 765 | 1 | 0.762 | 0.774 |
| d 567 | 1 | 0.784 | 0.859 |
| d 731 | 1 | 0.534 | 0.92 |
| Mean | 1 | 0.6964 | 0.8459 |
| Std. Dev. | 0 | 0.1614 | 0.07547 |

**Table F: Raw data to Fig BE in S1 Appendix – PBL proliferation (CellTrace_low_) [%]**

| blood donor | uninfected | uninfected + Ado | uninfected + 3'AMP | L.major DsRed | L.major DsRed + Ado | L.major DsRed + 3'AMP |
| --- | --- | --- | --- | --- | --- | --- |
| d 839 | 1,28 | 1,23 | 1,39 | 15,6 | 10,3 | 12,3 |
| d 915 | 2,09 | 1,51 | 2,43 | 17,7 | 5,74 | 12,4 |
| d 252 | 1,12 | 1,21 | 1,62 | 10 | 7,52 | 8,49 |
| d 269 | 3,04 | 4,76 | 4,91 | 24,8 | 18,5 | 23,3 |
| d 657 | 1,79 | 7,6 | 3,24 | 9,88 | 7,03 | 8,31 |
| d 018 | 1,55 | 1,24 | 2,29 | 16,1 | 14,5 | 13,8 |
| d 398 | 3,21 | 6,11 | 4,43 | 22,7 | 17,9 | 21,1 |
| d 765 | 3,44 | 2,62 | 5,58 | 23,9 | 18,2 | 18,5 |
| d 567 | 2,4 | 1,9 | 2,46 | 10,4 | 8,15 | 8,93 |
| d 731 | 1,19 | 1,41 | 2,2 | 7,76 | 4,14 | 7,14 |
| Mean | 2.111 | 2.959 | 3.055 | 15.88 | 11.2 | 13.43 |
| Std. Dev. | 0.873 | 2.344 | 1.439 | 6.328 | 5.565 | 5.724 |

**Table G: Supplemental data to Fig 7 - regression analyses**

| **Cell cycle** | p1/s1 3'flox | E10 iKO | F4 iKO | addback untagged |
| --- | --- | --- | --- | --- |
| [Inhibitor] vs. response -- Variable slope (four parameters) |  |  |  |  |
| Best-fit values |  |  |  |  |
| Bottom | 5,335 | 5,374 | 4,856 | 3,920 |
| Top | 91,12 | 85,00 | 88,31 | 94,52 |
| IC50 | 18,52 | 14,12 | 10,93 | 15,23 |
| HillSlope | 8,106 | 6,678 | 2,786 | 9,282 |
| logIC50 | 1,268 | 1,150 | 1,039 | 1,183 |
| Span | 85,79 | 79,63 | 83,45 | 90,60 |
| 95% CI (asymptotic) |  |  |  |  |
| Bottom | 2,429 to 8,241 | 0,8506 to 9,898 | 0,000 to 10,15 | 2,187 to 5,652 |
| Top | 84,04 to 98,21 | 77,39 to 92,62 | 77,42 to 99,19 | 91,51 to 97,54 |
| IC50 | 16,59 to 20,46 | 12,73 to 15,50 | 8,885 to 12,97 | 14,77 to 15,70 |
| HillSlope | 4,689 to 11,52 | 2,345 to 11,01 | 1,504 to 4,068 | 4,197 to 14,37 |
| logIC50 | 1,220 to 1,311 | 1,105 to 1,190 | 0,9487 to 1,113 | 1,169 to 1,196 |
| Span | 78,04 to 93,54 | 70,45 to 88,81 | 70,57 to 96,34 | 86,87 to 94,33 |
| Goodness of Fit |  |  |  |  |
| Degrees of Freedom | 36 | 35 | 34 | 33 |
| R squared | 0,9589 | 0,9203 | 0,9260 | 0,9909 |
| Sum of Squares | 1999 | 3712 | 3303 | 502,6 |
| Sy.x | 7,451 | 10,30 | 9,856 | 3,903 |

| **Phsophatidylserine exposure** | p1/s1 3'flox | E10 iKO | F4 iKO | addback untagged |
| --- | --- | --- | --- | --- |
| [Inhibitor] vs. response -- Variable slope (four parameters) |  |  |  |  |
| Best-fit values |  |  |  |  |
| Bottom | 6,006 | 6,865 | 9,809 | 10,20 |
| Top | 92,80 | 93,03 | 97,31 | 100,8 |
| IC50 | 8,107 | 9,833 | 8,692 | 13,37 |
| HillSlope | 3,382 | 3,776 | 2,365 | 3,594 |
| logIC50 | 0,9089 | 0,9927 | 0,9391 | 1,126 |
| Span | 86,79 | 86,16 | 87,51 | 90,62 |
| 95% CI (asymptotic) |  |  |  |  |
| Bottom | 0,000 to 12,07 | 0,4992 to 13,23 | 2,946 to 16,67 | 6,886 to 13,51 |
| Top | 83,93 to 101,7 | 83,16 to 102,9 | 83,82 to 110,8 | 93,48 to 108,2 |
| IC50 | 6,945 to 9,269 | 8,414 to 11,25 | 6,706 to 10,68 | 12,07 to 14,68 |
| HillSlope | 1,867 to 4,898 | 1,738 to 5,814 | 1,087 to 3,643 | 2,594 to 4,593 |
| logIC50 | 0,8417 to 0,9670 | 0,9250 to 1,051 | 0,8264 to 1,028 | 1,082 to 1,167 |
| Span | 75,64 to 97,94 | 73,82 to 98,51 | 71,51 to 103,5 | 82,05 to 99,19 |
| Goodness of Fit |  |  |  |  |
| Degrees of Freedom | 36 | 36 | 30 | 34 |
| R squared | 0,9137 | 0,8969 | 0,9086 | 0,9703 |
| Sum of Squares | 4860 | 5694 | 3858 | 1408 |
| Sy.x | 11,62 | 12,58 | 11,34 | 6,436 |

| **MTT assay** | p1/s1 3'flox | E10 iKO | F4 iKO | addback untagged |
| --- | --- | --- | --- | --- |
| [Inhibitor] vs. response -- Variable slope (four parameters) | Hit constraint | Hit constraint | Hit constraint | Hit constraint |
| Best-fit values |  |  |  |  |
| Bottom | ~ 6,787e-006 | ~ 4,577e-005 | ~ 6,241e-005 | ~ 8,097e-006 |
| Top | 1,094 | 0,9944 | 0,8127 | 1,003 |
| IC50 | 6,914 | 8,217 | 6,450 | 8,753 |
| HillSlope | -5,936 | -3,202 | -3,087 | -10,78 |
| logIC50 | 0,8398 | 0,9147 | 0,8095 | 0,9421 |
| Span | ~ 1,094 | ~ 0,9943 | ~ 0,8126 | ~ 1,003 |
| 95% CI (asymptotic) |  |  |  |  |
| Bottom |  |  |  |  |
| Top | 0,9820 to 1,205 | 0,9057 to 1,083 | 0,7435 to 0,8819 | 0,8505 to 1,156 |
| IC50 | 6,000 to 7,829 | 6,764 to 9,670 | 5,347 to 7,553 | 7,483 to 10,02 |
| HillSlope | -9,838 to -2,034 | -4,947 to -1,456 | -4,765 to -1,408 | -20,99 to -0,5694 |
| logIC50 | 0,7782 to 0,8937 | 0,8302 to 0,9854 | 0,7281 to 0,8781 | 0,8741 to 1,001 |
| Span | ??? | ??? | ??? | ??? |
| Goodness of Fit |  |  |  |  |
| Degrees of Freedom | 23 | 23 | 23 | 20 |
| R squared | 0,9130 | 0,9230 | 0,9344 | 0,8299 |
| Sum of Squares | 0,6171 | 0,3829 | 0,2268 | 1,048 |
| Sy.x | 0,1638 | 0,1290 | 0,09931 | 0,2289 |

**Table H: Raw data to Fig 8A – Infection rate (DsRed-positive) [%]**

| blood donor | L. major parental | L. major E10 iKO | L. major F4 iKO |
| --- | --- | --- | --- |
| d 435 | 79,9 | 89,2 | 87,5 |
| d 462 | 64,3 | 66,5 | 64,6 |
| d 162 | 86,2 | 82,3 | 88,5 |
| d 752 | 82,6 | 75 | 79,9 |
| d 767 | 75,6 | 66,8 | 65 |
| d 803 | 69,9 | 71,1 | 76,9 |
| Mean | 76.42 | 75.15 | 77.07 |
| Std. Dev. | 8.198 | 9.05 | 10.48 |

**Table I: Data to Fig 8B – Parasite burden, normalized to untreated**

| blood donor | L. major parental | L. major p1/s1 iKO E10 | L. major p1/s1 iKO F4 |
| --- | --- | --- | --- |
| d 435 | 1 | 0.85 | 1.04 |
| d 462 | 1 | 1.08 | 1.29 |
| d 162 | 1 | 0.74 | 0.91 |
| d 752 | 1 | 1.07 | 1.09 |
| d 767 | 1 | 0.95 | 1.01 |
| d 803 | 1 | 0.86 | 0.94 |
| Mean | 1 | 0.925 | 1.047 |
| Std. Dev. | 0 | 0.134 | 0.136 |

**Table J: Raw data to Fig 8C and Fig FB in S1 Appendix – PBL proliferation (CellTrace_low_) [%]**

| blood donor | unin-fected | un-infected + Ado | un-infected + 3'AMP | L. major parental | L. major parental + Ado | L. major parental + 3'AMP | L. major E10 iKO | L. major E10 iKO + Ado | L. major E10 iKO + 3'AMP | L. major F4 iKO | L. major F4 iKO + Ado | L. major F4 iKO + 3'AMP |
| --- | --- | --- | --- | --- | --- | --- | --- | --- | --- | --- | --- | --- |
| d 435 | 1,39 | 1,04 | 1,57 | 5,88 | 5,94 | 5,8 | 4,45 | 3,65 | 5,08 | 3,19 | 4,14 | 5,67 |
| d 462 | 1,56 | 1,4 | 1,96 | 14,2 | 14 | 12,4 | 12,4 | 12,7 | 12,3 | 11,6 | 11,3 | 13,3 |
| d 162 |  |  |  | 14,7 | 9,95 | 11,3 | 16,5 | 12,4 | 13,7 | 13,7 | 9,35 | 12 |
| d 752 |  |  |  | 12,9 | 12 | 10,1 | 14,8 | 14,1 | 16,1 | 12,3 | 8,84 | 12 |
| d 767 | 4,23 | 4,02 | 5,08 | 14,2 | 10,3 | 12,6 | 17,6 | 10,7 | 12,6 | 14,5 | 9,16 | 9,73 |
| d 803 | 7,64 | 4,49 | 5,96 | 12,7 | 10 | 12,3 | 14,6 | 11,6 | 15,9 | 15,1 | 11,9 | 15,6 |
| Mean | 3.705 | 2.738 | 3.643 | 12.43 | 10.37 | 10.75 | 13.39 | 10.86 | 12.61 | 11.73 | 9.115 | 11.38 |
| Std.  Dev. | 2.928 | 1.769 | 2.203 | 3.305 | 2.675 | 2.599 | 4.728 | 3.71 | 4.025 | 4.386 | 2.735 | 3.394 |

**Table K: Data to Fig 8D – PBL proliferation, normalized to untreated**

| blood donor | L. major parental | L. major parental + Ado | L. major parental + 3'AMP | L. major E10 iKO | L. major E10 iKO + Ado | L. major E10 iKO + 3'AMP | L. major F4 iKO | L. major F4 iKO + Ado | L. major F4 iKO + 3'AMP |
| --- | --- | --- | --- | --- | --- | --- | --- | --- | --- |
| d 435 | 1 | 1.01 | 0.986 | 1 | 0.82 | 1.142 | 1 | 1.298 |  |
| d 462 | 1 | 0.986 | 0.873 | 1 | 1.024 | 0.992 | 1 | 0.974 | 1.147 |
| d 162 | 1 | 0.677 | 0.769 | 1 | 0.752 | 0.83 | 1 | 0.682 | 0.876 |
| d 752 | 1 | 0.93 | 0.783 | 1 | 0.953 | 1.088 | 1 | 0.719 | 0.976 |
| d 767 | 1 | 0.725 | 0.887 | 1 | 0.608 | 0.716 | 1 | 0.632 | 0.671 |
| d 803 | 1 | 0.787 | 0.969 | 1 | 0.795 | 1.089 | 1 | 0.788 | 1.033 |
| Mean | 1 | 0.8525 | 0.8778 | 1 | 0.8253 | 0.9762 | 1 | 0.8488 | 0.9406 |
| Std. Dev. | 0 | 0.1414 | 0.09051 | 0 | 0.1479 | 0.1685 | 0 | 0.2501 | 0.1798 |

**Table L: Data to Fig 8G – Viable *L. major* from neutrophil killing assay, normalized to respective control without neutrophils**

| blood donor | parental +CytD | parental +CytD +DNase | E10 iKO +CytD | E10 iKO +CytD +DNase | F4 iKO +CytD | F4 iKO +CytD +DNase |
| --- | --- | --- | --- | --- | --- | --- |
| d 384 | 91.3 |  | 65.2 |  | 70.4 |  |
| d 507 | 79.17 | 100 | 62.5 | 94.74 | 68.75 | 79.17 |
| d 560 | 100 | 72 | 75 | 78.95 | 78.13 | 129.17 |
| d 361 | 81.25 | 110 | 41.67 | 86.21 | 52.94 | 85.71 |
| d 612 | 100 | 105 | 63.89 | 79.31 | 61.76 | 103.57 |
| Mean | 90.34 | 96.75 | 61.65 | 84.80 | 66.40 | 99.41 |
| Std. Dev. | 9.94 | 17.00 | 12.20 | 7.42 | 9.51 | 22.36 |

**Supplemental methods:**

**Determination of metacyclic promastigotes**

2x10^8^ *L. major* parasites were incubated in 500 µl RPMI +100 µg/ml peanut (*Arachis hypogaea*) lectin (SigmaAldrich) for 30 min at room temperature. After centrifugation for 10 min at 200 x*g*, the supernatant containing the metacyclic promastigotes was washed in DMEM +20 mM galactose before counting.

**Whole genome sequencing:**

Genomic DNA was isolated using the Quick-DNA Miniprep Plus Kit (Zymo Research) from the parental and *p1/s1* knockout *L. major*. One µg genomic DNA per condition was ultrasonicated to an average of 300 bp using the M220 Focused-ultrasonicator using micro TUBE-50 cuvettes (Covaris). DNA ends were prepared for ligation with NEBNext® Ultra™ II End Repair/dA-Tailing Module (NEB) following the protocol of the manufacturer. After purification with one volume of magnetic beads the DNA was ligated with 20 pmol of custom double stranded Y-linkers (for the oligonucleotides see Table M: Primer sequences) using the Blunt/TA Ligase Master Mix (NEB) for 15 minutes at room temperature. The bead purified ligation reactions were subjected to qPCR to determine the necessary PCR cycle numbers for barcoding PCRs, which were performed with the NEBNext® High-Fidelity 2X PCR Master Mix (NEB) using the *PE_nest_IndN* and *Illumina1* primers with the following cycling conditions: 98 °C 30s; 6 cycles of 98 °C 10 s, 65 °C 75 s; 65 °C 5 min. The PCR products were magnetic bead purified and sequenced with single end 1 x 132 bp setting on a NextSeq2000 Instrument (Illumina).

**Data availability:**

The raw sequence files of the whole genome sequencing of the parental and the genome edited strains are available as SRA data at NCBI BioProject: PRJNA1330523

(<https://www.ncbi.nlm.nih.gov/bioproject/PRJNA1330523>).

**NGS read processing:**

Bioinformatic processing was conducted within a reproducible Conda environment to ensure consistency in software versions and dependencies. Raw whole-genome sequencing (WGS) data for *Leishmania spp.* were analyzed using a modular workflow consisting of genome retrieval, read preprocessing, alignment, genome polishing, and mapping complexity characterization.

Raw sequencing reads were processed with Trim Galore! (v0.6.10), which utilizes Cutadapt for adapter removal and base quality trimming. Post-trimming quality control was conducted using FastQC (v0.12.1). Processed reads were aligned to the reference genome for Leishmania major strain Friedlin (NCBI GenBank accession: GCF_000002725.2) using BWA-MEM2, followed by sorting and indexing with SAMtools (v1.18).

Reference-guided genome polishing was performed using Pilon (v1.24), which incorporates aligned read information to detect and correct single nucleotide polymorphisms (SNPs), insertions, and deletions (indels). Variant call format (VCF) files were generated during polishing and filtered to retain high-confidence, non-reference variants alongside browsable extensible data (BED) containing CNV calls. These Pilon-derived variants were subsequently incorporated into downstream analyses, including copy number inference and gene-level annotation.

**Copy number variation analysis:**

To identify regions with significant copy number changes, a differential expression-like analysis was performed using DESeq2 (v1.48.1). Pilon-derived copy number estimates were treated as count-like input for DESeq2, with comparisons defined by sample group (parent vs. clone). Copy numbers were modeled under a negative binomial framework with treatment groups defined by sample origin (parental vs. derived). Normalization was performed via size factor estimation, and log2 fold changes were stabilized using apeglm-based shrinkage and significance was assessed using a threshold of absolute log₂ fold-change ≥ 1 and adjusted p-value < 0.05.

Genomic regions were mapped to annotated genes using coordinate overlap, and gene-level copy number summaries were calculated. Gene biotypes and mapping complexity (e.g., multimapping proportion) were integrated to annotate and interpret CNV patterns.

All CNV predictions were visually inspected using Integrative Genomics Viewer Web App (IGV-Web) (v2.2.8) to confirm consistency with read coverage and identify potential artifacts or complex structural variation. Visualization of inter-sample CNV patterns was performed using ggplot2 (v3.5.2). Final differential results were exported for integration with downstream genomic analyses and candidate gene prioritization.

**Table M: Primer sequences for whole genome sequencing**

| PE_nest_indN | CAAGCAGAAGACGGCATACGAGATNNNNNNGTGACTGGAGTTCAGACGTGTGCTCTTCCGATCT (Ns stand for TruSeq barcodes) |
| --- | --- |
| Illumina 1 | AATGATACGGCGACCACCGAGATCTACACTCTTTCCCTACACGACGCTCTTCCGATCT |
| Y-linker+ | gtaatacgactcactatagggctccgcttaagggacTCAGACGTGTGCTCTTCCGATC*T |
| Y-linker- | GATCGGAAGAGCACACG-SpacerC3 |

**Homology modelling of p1/s1:**

To determine whether proteins coded in the *LmJF.30.1460-1510* cluster possess the active site characteristic of p1/s1 nucleases and potentially exhibit nucleotidase activity, a template search was performed using the SWISS-MODEL server [74]. The search returned several nuclease enzymes as structural homologs, including Q9C9G4 from *Arabidopsis thaliana*, P24021 from *Aspergillus oryzae*, Q0KFV0 from *Solanum lycopersicufrom*, and P24289 *Penicillium citrinum*, all with experimentally determined 3D structures.

Superimposition of the AlphaFold-predicted structures of Q4Q7F3 and Q4Q7F4 onto homologous structures revealed that both proteins have an active site homologous to that of known nucleases (Figures 2C and D). Among them, the bi-functional nuclease AtBFN2 from *Arabidopsis thaliana* (PDB ID: 3W52) showed the highest similarity in terms of conserved active site residues to Q4Q7F3 and Q4Q7F4. Notably, Q4Q7F3 and Q4Q7F4 differ by only four amino acid residues. A signal peptide was present in both sequences but was removed prior to superimposition. Also, sequence and structural alignments indicated that Q66VY6, Q4QGQ3, and Q4Q630 possess the conserved active site.
