# Supplementary material for: p1/s1, a 3’-nucleotidase/nuclease, allows Leishmania major to circumvent host innate immune response mechanisms
Source: PLoS Pathog. 2026 May 20;22(5):e1014197. doi: 10.1371/journal.ppat.1014197 (PMC13232948; doi:10.1371/journal.ppat.1014197)
Supplement: Attachment — Submitted filename: 160725_1_rebuttal_3673529_t4prh3.docx [file ppat.1014197.s004.docx]

**Reviewer #1**

Evidence, reproducibility and clarity

*In this manuscript the authors describe a comprehensive analysis of the role of p1/s1 nucleotidases/nucleases have in modulated the immune response to Leishmania. They show that the parasite can break down 3' AMP to generate adenosine that dampens the immune response. Plus, they show that this family of nucleotidases/nucleases is important for breaking down NETs released by neutrophils. The deletion of a large portion of the genome using the inducible CRE-lox system was an elegant approach. Moreover, they demonstrated that the loss of this locus was associated with an increase in expression of proteins associated with purine salvage. Overall, the manuscript is well-written and clear, with the data presented supporting the conclusions made.*

*The assays for nucleotidase and nuclease activity rely on incubating the parasites in a buffer for 1 or 3 hours before separating into a pellet versus supernatant fraction. While the buffer for nucleotidase assay is at pH similar to culture conditions with glucose present the one for the nuclease assay is at a lower pH normally associated with amastigotes without a source of energy. Were the cells still alive after incubation in this buffer? Would they grow out at a normal rate if returned to culture medium? On a related note, does the actual assay kill the cells or not? I would like the authors to clarify whether the activity of the pellet is that seen from enzymes on the surface of the parasite or just the enzyme activity from the cell as a whole.*

We thank for the legitimate questions. Visual inspection by microscopy confirmed that incubation of promastigotes in the nuclease reaction buffer for 3 hours plus the regarding reaction time does not comprise parasite viability or membrane integrity over the full course of the assay. Therefore, the pellet fraction represents membrane-bound ecto-enzymatic activity. A clarifying addition was made in the corresponding Methods section:

Lines 192-193: “Intact pelleted parasites represented membrane-bound ecto-nucleotidase activity, the supernatant fraction secreted ecto-nucleotidase activity.”

Lines 204-205: “Intact pelleted parasites represented membrane-bound ecto-nuclease activity, the supernatant fraction secreted ecto-nuclease activity.”

*While not necessary for this manuscript additional cell lines in which just either the secreted or membrane bound enzymes were deleted might add greater understanding to their specific roles in controlling the immune response.*

We thank for the reasonable suggestion. Generating such strains was not feasible due to the extremely high homology between the coding sequences and intergenic regions at potential 5’ sgRNA binding sites. Thus, specific sgRNAs targeting either the secreted or membrane-bound enzymes for CRISPR/Cas 9-mediated deletion could not be designed.

*The authors state it is the ecto/secreted activity that is more important but then perhaps the authors could then speculate on the roles of the membrane bound nucleases?*

Many thanks for this advice. We now discussed a potential role of the membrane-bound nucleases in the text.

Lines 745-746: “These class I nucleases might only participate in extracellular nucleic acid degradation for nutrient acquisition and NET escape. “

*Minor comments
Line 87 - deletion instead of depletion*

Correction was implemented.

*Line 338 - I was confused by the terminology of sublethal doses that lead to cell death. It is not sublethal if it kills the cells. I understand that it is the speed of killing but I think a different term would be more appropriate*.

The term was replaced by more appropriate descriptions like low-dose treatment .

Lines 367-371: “In order to designate such proteins in *L. major*, we applied stress to the parasites by treatment with low doses of miltefosine, an antileishmanial drug, or staurosporine, an unspecific kinase inhibitor, that would lead to cell death after 24 hours. Following treatment with miltefosine for 3 hours or staurosporine for 6 hours, with early cell death processes being induced (SuppFig. 1), we performed a quantitative proteome analysis to compare protein expression.”

*In figure 1A-C why is there a gap between the highly enriched or depleted proteins? Is this because these proteins were undetected in the other sample so they've been giving an arbitrary value so they can be plotted?*

Yes, this is correct. In order to plot proteins that were exclusively detected in only one condition, we set an arbitrary value (similar to a minimum value imputation). We apologize if this was not clearly stated in the manuscript. We now mention this in the figure legend and also in the material and methods section.

Material and Methods, lines 179-180: In order to plot proteins that were exclusively detected in only one condition, we set an arbitrary value.

Figure legend lines 355-357: “Visible gaps in the fold change separate protein hits that revealed increased abundance in one condition but had not been detected in the other condition. In order to plot these proteins, we set an arbitrary value for each undetected protein.

Results text lines 371-374: “Volcano plots of proteins that show significantly increased abundance in the treated or untreated conditions are displayed in Fig1A-B. Here, proteins with increased abundance in one condition that is displayed by a segregated log2 fold change, had not be detected in the other condition.”

*Figure 2H - can you provide more detail in the legend or on the gel, not clear what Lm is or what is in the first lane of the gel. Plus, I think the box is not in correct position as it is not over the M13 DNA.*

We thank for the good observation. The box position was corrected and the figure description completed with information on the labeling.

Line 418-419: “Representative gel of **G**. Box denotes area of semi-quantification. dsM13: undigested product; *Lm*: control with parasites but no substrate; DNase: Substrate incubated with DNase, first and last lane: DNA ladder.“

*Line 377 - were all the detected peptides from the conserved region of these proteins? If so why in table 1 is the top protein Q4Q7F3 which is one of the secreted enzymes and not any of the others?*

We thank for the good observation. Clarifying details were added to table 2 and its description.

Lines 394-395: “All peptides detected for p1/s1 could be assigned to the conserved C-terminal region of proteins in the cluster (Fig. 2A). “

*Line 404 - should be corresponding well to*

Has been corrected accordingly.

*Figure 2D/E I found it hard to differentiate between the pink and red dots can you change the colour scheme*

Fig 3D/ E, Fig 8A-D and SupFig. 2C-E have been changed accordingly to a color scheme easier to differentiate between the infected and infected + 3’-AMP conditions.

*Figure S2C is not mentioned in the main text. Plus, could the authors clarify in the S2C figure legend that this differs from Fig 2B as it does not have PBLs present it took some time for me to work that out.*

Many thanks for noticing the missing description. Due to figure adjustments as recommended by the different reviewers, we decided to delete SupFig. 2C from the manuscript as it does not provide additional information. Instead, as suggested, we now show absolute values of PBL proliferation upon macrophage infection with Leishmania and supplementation with either adenosine or 3’AMP in SupFig. 2D.

*For the growth analysis in Fig 4F what density were these cultures seeded at?*

A clarifying statement was added to the methods section.

Lines 98-99: “Cultures were seeded on day 0 with 50,000 viable *L. major* in 100 µl per well of a 96-well plate .”

*Line 557 - volcano plot*

Has been changed accordingly.

*In Fig S6 there did not seem to be many reads to the nucleotide transporter, is this gene actually present in the genome, as there are not many reads over the 5' end of the gene?*

We can explain this as follows. In the figure, there is nearly constant read coverage across the displayed regions. However, there are “white” reads that have a mapping quality of 0, indicating that they align equally well to multiple genomic loci and therefore cannot be assigned uniquely. These reads align without mismatches at the displayed location, but the same alignment quality is obtained elsewhere in the genome. This suggests the presence of identical or near-identical sequence elsewhere, such as a paralogous gene, duplicated region, or shared repetitive sequence, rather than uniquely supporting this locus. We have not determined these alternative loci.

Significance

*Overall, this is a comprehensive study of the role of p1/s1 nucleotidases/nucleases have in modulated the immune response to Leishmania that combines a broad range of approaches. The experiments are well-controlled and appropriately described. This manuscript provides for the first time a specific test of the function of these genes through the use of gene deletion. Hence it should have broad interest from those studying fundamental parasite cell biology to those studying translational to clinical approaches.

I am a molecular parasitology studying fundamental leishmania cell biology.*

**Reviewer #2**

Evidence, reproducibility and clarity

*Schmelzle and colleagues have investigated the role of p1/s1, a 3-nucleotidase/nuclease produced by Leishmania major, by generating a deletion mutant through a clever approach involving Cas9T7 RNA polymerase and a dimerizable Cre. Their main findings are that the p1/s1 deletion L. major mutant is susceptible to NETosis but remains as susceptible to miltofosine than the parental strain and the add-bac. Moreover, the authors provided evidence that p1/s1 is used by Leishmania to generate extracellular adenosine to suppress inflammatory cytokine secretion and to reduce the lymphocyte proliferation in a human primary cell model.

The generation of a p1/s1 deletion mutant has been conducted elegantly. Using this mutant, the authors have validated previous findings that this enzyme is required for Leishmania to degrade NETs. In the case of miltefosine susceptibility, their data differs from those of a previous study using a natural L. infantum deletion mutant. Overall, this an interesting study. A few issues deserve to be addressed.*

*1- In Figures 3B and 8B, the authors measure the fate of Leishmania inside macrophages as "relative parasite burden". It is preferable to show the real numbers, such as the number of parasites/100 infected macrophages, from t=0 to t= day 4. Otherwise, it is impossible to determine whether the parasites replicate at different rates or are eliminated at different rates, compared to control groups.*

We thank for the constructive feedback to enhance the infection data. We chose to quantify macrophage infection with *L. major* in a flow cytometry-based detection method utilizing DsRed expressing strains or anti-*Lm* antibodies as this method can easily be applied to assess more than 10,000 cells. Thus, this methods analyzes a more representative sample than microscopic counting of intracellular parasites and reduces human biases in analysis. However, this method does not provide absolute parasite counts per macrophage as it is based on the mean fluorescence intensity of the parasite-specific DsRed signal. We have added the non-normalized DsRed MFI, corresponding to parasite burden, in supplemental figure 2 panel D. As data in both, Fig 3B and 8B, did not show significant differences in terms of parasite burden, no experiments surveilling infection over time were conducted.

*2- Figure 2A, it would be a nice addition to include an inert phagocytic prey and/or LPS to assess the impact of Ado and 3'-AMP on TNF secretion.*

We thank for the constructive feedback to improve the strength of Fig 3A. An LPS control was included during development of the experimental setup as positive control for TNF secretion but detected cytokine levels were far beyond the upper detection limit of the ELISA method. Thus, this control was not included in the final setup.

The addition of a phagocytic bait could indeed enhance this data set. However, we did not include such a control in the setup as the presence of Ado or 3’-AMP did not influence the basal TNF secretion of uninfected macrophages.

Significance

*The strength of this study is the approach used to generate a deletion mutant of p1/s1. This mutant was then instrumental in validating/invalidating previous studies on the role of the Leishmania 3'-nucleotidase/nuclease.

Whereas in vitro analyses suggest that p1/s1 is a pivotal factor during infection, this study would be more conclusive with experimental infection in a mouse model of cutaneous leishmaniasis.

The audience of this research is rather specialized, and would interest researchers working on basic aspects of Leishmania pathogenesis.*

**Reviewer #3**

Evidence, reproducibility and clarity (Required):
Summary:
*The manuscript "p1/s1, a 3'-nucleotidase/nuclease, allows Leishmania major to circumvent host innate immune response mechanisms" by Schmelzle et al. presents a comprehensive investigation of a Leishmania major 3'-nucleotidases/nuclease in parasite infectivity and immune evasion, employing quantitative proteomics, immunological profiling using primary human cells, and Cas9-mediated conditional null mutant analysis using a dimerizable Cre system. The authors identify a class I nuclease showing increased abundance under Miltefosine and Staurosporine A treatment, and demonstrate its growth phase-regulated expression observing upregulation in stationary but not logarithmic promastigotes, as well as its ecto-nucleotidase and -endonuclease activities using purified protein in activity assays. Functional studies show that supplementing infected, IFNγ-activated human monocyte-derived macrophages (hMDMs) with external adenosine or 3'-AMP decreases TNFα secretion and suppresses proliferation of autologous peripheral blood lymphocytes, indicating immune dampening. Using nucleotidase-deficient parasites targeting the p1/s1 cluster, the authors further link reduced nucleotidase activity to (i) diminished adenosine-mediated anti-inflammatory responses (as assessed by lymphocyte proliferation assays), and (ii) increased susceptibility to neutrophil extracellular traps (NETs). Collectively, these data provide robust evidence confirming the role of Leishmania extracellular nucleotidases in immune escape from NETs and immune subversion via enhanced anti-inflammatory signaling driven by extracellular 3'-AMP/adenosine.*


Major comments:
*Comment 1: While the manuscript is generally well written, the figure legends lack sufficient detail to enable readers to fully interpret the results independently. Specifically, the legends should include: (i) a brief description of the type of analysis performed, (ii) concise methodological information, (iii) definitions of all abbreviations and symbols used in the figures, and (iv) clear identification of controls. As currently presented, readers must repeatedly cross-reference the Methods and Results sections to understand the figures, which disrupts the flow of comprehension. Figures and their legends should be self-contained and interpretable without requiring extensive navigation through other sections of the manuscript.*

We thank for the constructive comment to improve the manuscript’s readability. The manuscript has been changed accordingly with extended figure legends.

*Comment 2: The reviewer is not convinced by the rationale provided for using the proteomics analysis in Figure 1 to justify the downstream focus on p1/s1, which is only one of nine proteins found to be upregulated in response to three forms of sub-lethal stress. Given the established link between Leishmania nucleotidases and parasite viability and immune evasion, there is already sufficient justification for investigating the nucleotidase cluster on chromosome 30, particularly the secreted p1/s1 protein*.

We thank for the critical questioning regarding the identification of p1/s1 as protein of interest. Our intention for the mass spectrometry approach was to reveal proteins that are involved in Leishmania cell death regulation. As a result of this analysis, p1/s1 was identified as one out of nine proteins hits, that were found in increased levels upon cell death induction or intrinsic cell death in the statPh. Especially, it was one out of four proteins exclusively detected in cell death conditions while lacking detection in untreated or logPh parasites. Given the known involvement of Leishmania cell death induction for parasite infectivity (van Zandbergen et al., 2006), we were curious whether p1/s1 with its dual nucleotidase/nuclease function might be a key regulator of cell death which, of course, could affect cell viability and immune evasion. The respective results section was adapted to clarify this derivation.

Lines 385-387: “One of the exclusively detected hits, p1/s1, is the only protein with predicted enzymatic activity, namely a class I nuclease, and with proposed involvement in host-parasite interactions.”

Therefore, this proteome data set suggests a potential link of p1/s1 to parasite cell death and, potentially DNA fragmentation seen during this process. However, this hypothesis was not supported by data on cell death hallmarks presented in Fig. 7.

*Additionally, the presentation of the proteomics data is confusing:
(i) For each volcano plot, two groups of differentially expressed proteins are visible: one close to the Y-axis (representing proteins detected in both conditions with abundance differences) and another further out with high log2-fold changes. What do these proteins represent-are they uniquely detected in only one condition? If so, why is there such a wide dispersion? Shouldn't these theoretically show infinite fold change?*

Very good point. Explanation of the wide dispersion of hits was added to the text, the figure description and to the methods section, to explain that these gaps indeed separate proteins that are enriched/depleted and proteins that were only detected in one of the conditions within the comparison.

Figure legend lines 355-357: “Visible gaps in the fold change separate protein hits that revealed increased abundance in one condition but had not been detected in the other condition. In order to plot these proteins, we set an arbitrary value for each undetected protein.

Results text lines 371-374: “Volcano plots of proteins that show significantly increased abundance in the treated or untreated conditions are displayed in Fig1A-B. Here, proteins with increased abundance in one condition that is displayed by a segregated log2 fold change, had not be detected in the other condition.”

*(ii) The inclusion of stationary-phase promastigotes as an additional form of sub-lethal stress is problematic. According to Fig S1, 50% of these parasites are dead or undergoing cell death at this phase. How does this impact the validity of the analysis?*

Thanks for giving us the opportunity to make the comparison between drug treatment and stationary phase more comprehensive.

The term „sub-lethal“ drug concentration was now replaced by more appropriate descriptions like ‘low-doses’ of drugs to clarify that the drug treatments for 3-6 hours, as used for the mass spec samples, induce cell death processes that would execute cell death if treated for 24 hours (see SupFig. 1).

The stationary phase of promastigotes, as we culture it on semi-solid blood agar plates in our lab, contains approximately 50-60% dead parasites (see SupFig. 1). Of course, this death is not due to a specific treatment but only parasite proliferation followed by nutrient restriction. However, also this stationary phase reflects a stress condition upon which the parasites induce their own apoptosis.

Assessing the quantified proteomes of the different cell death-linked conditions compared to the viable logarithmic phase control allowed us to identify proteins potentially involved in parasite cell death, while being able to filter out proteins that might just reflect drug-specific responses. Thus, analysis of parasites that induced cell death via different triggers, in our opinion, makes the obtained mass spectrometry results more valid and robust.

The definition of the term ‘stationary phase’ used in this manuscript is now being explained the methods section.

Lines 99-101: “Logarithmic-phase (logPh) promastigotes, 3 days post passaging, or stationary-phase (statPh) promastigotes from 6-8 days post passaging, consisting of approximately 50-60% apoptotic-like and 2-10% metacyclic parasites, were used for experiments. ”

*(iii) Line 341: "Proteins involved in drug-specific responses or metacyclogenesis of the statPh are solely detected in single conditions and are thus excluded." Which specific proteins are these?*

In addition to the data repositories stated under “Data availability” in the Methods section, an Excel sheet detailing the detected protein hits has been added to the supplementary data so the interested reader can identify these proteins.

*Determining the proportion of metacyclic parasites is necessary to properly interpret the results. To what extent does the analysis of mixed parasite populations affect the results?*

We thank for the critical questions regarding our in vitro infection setup. We have data available that our stationary phase promastigotes contain 2-10% metacyclic promastigotes , as measured by PNA enrichment, which can be stably reproduced with our culture conditions. We generally do not purify metacyclic parasites for setting up an infection experiment as our infection system relies on the presence of apoptotic *Leishmania* within the virulent inoculum, which is known to affect the course of infection *in vitro* and *in vivo*.

(*iv) Line 352: ".... infectious statPh L. major...." is misleading. Stationary-phase promastigotes are generally considered non-infectious, except for the small subset of metacyclic parasites, which are truly infectious. The authors use statPh parasites in one context as a model for cell death and then in another as a model to study infectivity in another, which is inconsistent and problematic*

We thank for the critical questions regarding the stationary phase in our culture system. Indeed, the Leishmania stationary phase, harvested upon 6-8 days culture on semi-solid blood agar plates, consists of approximately 50-60% apoptotic-like parasites (see SupFig. 1). In our opinion, this does not contradict the fact that the stationary phase is an infective population. Of course, the metacyclic parasites only account for a small subset in this population (2-10%). However, it is well accepted that the apoptotic parasites, as part of this population, are efficiently transmitted to and taken up by host cells, and affect the course of infection. Thus, the term ‘stationary phase’ describes the composition of the whole parasite population with different subsets affecting infection, and it is used opposing the non-infectious logPh culture lacking metacyclic and apoptotic promastigotes.

A more detailed description regarding the composition of a stationary phase in our culture system has now been added to the methods section in lines 99-101 (see above).

(*v) For Fig 1E: What is meant by 'enriched'? Is this equivalent to 'increased abundance'? Why are there so few 'enriched' proteins specific to individual conditions if the volcano plots display hundreds? Where are the excel tables for these analyses? Labeling the analyses according to only one condition is confusing and should be corrected to 'xxx versus yyyy'.*

Many thanks for making us aware of this lack of clarity. We agree that the term „enriched“ is a bit confusing in this context, and we now changed this expression throughout the text to ‘increased abundance’.

Table 2: Remarks to proteins: either “Not detected in viable control, exclusive to cell-death related conditions” or “Increased abundance in statPh *L. major* and upon cell death induction.”

Regarding the protein numbers in the Venn diagram: The sum of shown protein numbers in all sections for each comparison indeed results in >100 enriched proteins per condition, aligning with the displayed hits in the volcano plots.

*(vi) For Fig S1: How does the annexin V staining relate to the number of dead parasites? A FACS analysis of PI-stained parasites should also be presented. It seems that over 50% of stationary-phase promastigotes are undergoing cell death. If this is accurate, it suggests a serious issue with the culture conditions, as L. major typically shows no more than 5% dead parasites, even after prolonged stationary-phase culture.*

Many thanks for this comment. A definition of the stationary phase in our established culture system were added to the methods sections. Indeed, such a stationary phase harvested 6-8 days post passaging consists of approximately 50-60% apoptotic-like parasites.

AnnexinV staining of phosphatidylserine on the cell surface of Leishmania parasites is an established marker to confirm early apoptosis (e.g. shown in van Zandbergen, Ger, et al. "Leishmania disease development depends on the presence of apoptotic promastigotes in the virulent inoculum." *Proceedings of the National Academy of Sciences* 103.37 (2006): 13837-13842.)

To clarify this, the legend of SupFig. 1 has been changed accordingly:

“Phosphatidylserine exposure as cell death marker determined in flow cytometry by AnnexinV positivity of promastigotes treated with 25 µM miltefosine or 25 µM staurosporine”

*Comment 3: For Figure 2, the construction of the His6-tagged transgenic parasites needs to be thoroughly described in the Methods section. Given that proteins are continuously produced, it is surprising that no signal of newly synthesized, but not yet secreted, protein was detected in the lysate. This point should be addressed and discussed*.

We thank for the useful addition, the appropriate methodological description has now been added in lines 316-323:

“**Generation of *L. major* strains overexpressing p1/s1-His_6_:** *p1/s1* (*LmjF.30.1500*) was inserted into the multiple cloning site of pLexLm plasmid including an in-frame fusion with the encoded His_6-_tag. Identity of the insert as *LmjF.30.1500* was confirmed by Sanger sequencing. For generation of strain overexpressing *p1/s1*-His_s_, 60x10^6^ *L. major^Cas9/T7/^*eGFP logPh promastigotes were electroporated with SwaI (New England Biolabs) linearized and sterilized plasmid. To generate strains overexpressing *p1/s1*, a *L. major^Cas9/^*^T7/eGFP^ strain was used. Electroporated cells were selected and cultured as described above for *L. major* addback strains.”

The lack of p1/s1-His_6_ signal in the parasite lysate was added to the discussion in lines 702-706: “p1/s1, due to its N-terminal signal sequence, was found to accumulate as secreted enzyme in the supernatant of a promastigote culture. No protein was detected in the parasite lysate, likely due to immediate secretion after protein biosynthesis.”

*Comment 4: Why are TNFα levels so low in uninfected, IFNγ-activated hMDMs (Figure 3A)? Please clarify and explain, as infection with Leishmania clearly enhances TNFα secretion. This suggests that uninfected activated macrophages are only primed for TNFα production, which becomes upregulated upon infection*.

We are happy to answer your question.

Yes, you are right. IFNγ, which we use for polarization of human GM-CSF-differentiated monocyte-derived macrophages into an M1-like phenotype, only primes human macrophages. There is no substantial TNF alpha secretion upon IFNγ treatment alone but it can enhance TNFα secretion to subsequent stimuli, which, in our case, is the infection with Leishmania. This is expected and well described in publications by ourselves and others.

- Volkmar, Kerren, et al. "Investigating pyroptosis as a mechanism of L. major cell‐to‐cell spread in the human BLaER1 infection model." *Molecular Microbiology* 121.3 (2024): 453-469.;
- Berner MD, Sura ME, Alves BN, Hunter KW Jr. IFN-gamma primes macrophages for enhanced TNF-alpha expression in response to stimulatory and non-stimulatory amounts of microparticulate beta-glucan. Immunol Lett. 2005 Apr 15;98(1):115-22. doi: 10.1016/j.imlet.2004.10.020;
- F P Nestel, K S Price, T A Seemayer, W S Lapp; Macrophage priming and lipopolysaccharide-triggered release of tumor necrosis factor alpha during graft-versus-host disease. J Exp Med 1 February 1992; 175 (2): 405–413. doi: doi.org/10.1084/jem.175.2.405)

*Additionally, Line 438 ("Addition of 3'-AMP to the infection partially mimics the effect observed with adenosine.") is overstated; the lack of a significant difference should be highlighted. Consequently, the final statement on line 452 ("Thus, by generating adenosine through their 3'-nucleotidase activity, L. major can suppress an effective early immune response.") requires moderation.*

We thank for the critical remarks. The phrases have been adjusted to underline the limitation of the statements.

Lines 488-489: “Addition of 3’-AMP to the infection can mimic the effect observed with adenosine to some extent.”

*Comment 5: For Figure 4F, the authors note (line 493) that "both p1/s1 iKO strains show greatly reduced parasite density in the logPh and statPh compared to the parental strain." How does this growth defect affect the development of infectious metacyclic parasites, whose generation is density-dependent and can substantially impact infection results when using stationary-phase promastigotes? Is the reduced density associated with increased parasite mortality? This should be tested by FACS analysis of PI-stained cultures.*

We appreciate these justified remarks. Stationary phase cultures of all strains used for infections were only used if they had comparable proportion of dead parasites (approx. +/- 5%). If required, cultures of p1/s1 deficient strains were seeded a day earlier to reach comparable stationary phases at the day of the infection experiment. As a result, observed overall percentage of infected macrophages and other infection determinants did not vary (Fig. 8A-C). Employing peanut agglutinin (PNA) purification, we confirmed that stationary phase cultures of all strains contain between 2-10 % metacyclic promastigotes (data not shown).

Minor comments:
*Line 19: The statement "Employing quantitative proteomics, we identified a class I nuclease p1/s1 cluster in L. major....". is misleading. First, this cluster is known and has been already annotated; second, only one protein encoded by this cluster shows increased abundance in the stress-regulated proteome. This needs to be reformulated in a more precise manner.*

Thanks for this input. The listed proteins in Tab. 2 have been adjusted. To our knowledge, p1/s1 of *L. major* had not been characterized in terms of enzymatic activity before. The wording in line 19 has been corrected accordingly from ‘identified’ to ‘characterized’.

*Line 95: Methods. A description of the generation of molecular constructs and transgenic parasites for His6-tagged p1/s1 is missing.*

A paragraph containing the requested information has been added to the Methods section.

Lines 316-323 & see above.

*Line 98: "Logarithmic-phase (logPh) promastigotes, 3 days post passaging, or stationary phase (statPh) promastigotes, 6-8 days post passaging, were used for experiments". The initial inoculation density needs to be given in order to render this experimental set up reproducible for the reader.*

Thanks for noticing this lack of information. The inoculation density has been added to the methods section.

Lines 98-99: “Cultures were seeded on day 0 with 50,000 viable *L. major* in100 µl per well of a 96-well plate.”

*Line 222: Determination of hMDM infection. Why were the infections not performed with infectious, metacyclic promastigotes that can be easily purified from stationary culture by peanut/ricin lectin agglutination? How was the level of metacyclics controlled in the various infection experiments and strains used? How were differences in initial parasite uptake taken into account (e.g. were parasite numbers normalised to the initial uptake at 3h post-infection)? Were infection data normalized to parasite numbers at the initial uptake after 3h? The infectivity of the parasite should be validated plotting 'number of parasites/100 macrophages' over the 4 days infection period.*

We thank for the critical questions regarding our in vitro infection setup. We have data available that our stationary phase promastigotes contain 2-10% metacyclic promastigotes , as measured by PNA enrichment, which can be stably reproduced with our culture conditions. We generally do not purify metacyclic parasites for setting up an infection experiment as our infection system relies on the presence of apoptotic *Leishmania* within the virulent inoculum, which is known to affect the course of infection *in vitro* and *in vivo*. We did not generate data for initial parasite uptake. As both, Fig 3B and 8B, did not show significant differences in terms of parasite burden, no experiments were conducted to monitor infection over time. We chose to quantify macrophage infection with *L. major* in a flow cytometry-based detection method utilizing DsRed-expressing strains or anti-*Leishmania major* antibodies, as this method can easily be applied to assess more than 10,000 cells. Thus, this methods analyzes a more representative sample than microscopic counting of intracellular parasites and reduces human biases in analysis. However, this method gives no absolute parasites per macrophage values, as it is based on the mean fluorescence intensity of the parasite-specific signal.

*Line 235: 'Macrophages and PBLs were stained in 1 μM CellTrace Far Red....'. How was the effect of the staining on cell viability controlled for?*

Thanks for noticing the missing information. Only viable cells, as determined by viability staining, were included in the analysis. The share of viable lymphocytes was not comprised by Cell Trace staining and further cultivation. Statements to clarify this were added to the methods section.

Lines 245-246 and 258-259: “Cell viability was monitored using staining with 1:1000 ZombieAqua (BioLegend).”

*Line 430: "As shown in Fig. 2C,..;" - this is the wrong panel to cite here.*

The reference has been corrected accordingly.

*Line 435: "... whether macrophage exposure to adenosine or 3'-AMP during L. major infection can the pro-inflammatory cytokine response....' - there is a verb missing here.*

Thanks for the hint. The missing word “reduce” has been added.

*Line 480: The information shown in Tab. 3 is trivial. Either remove and indicate the info in the text, or move to supplementary data.*

Thank you for critically reviewing the presentation format. The table has been removed accordingly.

*Line 548: The authors point out that compensatory "....upregulation by genomic adaptation is unlikely. This rather points to a posttranscriptional regulation, as typical for Leishmania." Demonstrating this post-transcriptional mechanism using a simple RT-qPCR approach would strengthen this claim here and add depth to this analysis.*

We thank for the justified remark. We have conducted such an analysis for the redundant 3’-nucleotidases/nucleases and found no differential RNA levels in the knockout strains, see <https://doi.org/10.25358/openscience-13779>, Fig. 4.13 on page 94.

As previous publications showed similar results for other purine salvage proteins upon purine starvation ([65] Carter, Nicola S., et al. "Adaptive responses to purine starvation in Leishmania donovani." *Molecular microbiology* 78.1 (2010): 92-107.), we did not attempt RT-qPCR of transporter or other genes.

*Line 574: ".... pathways like DNA degradation, and does not play a role in miltefosine sensitivity of L. major." Tone this statement down as this could be masked by the shown compensatory response.*

The sentences has been toned down.

Lines 624-625: ”Thus, p1/s1 does not seem to be involved in intracellular cell death pathways like DNA degradation or miltefosine sensitivity of *L. major*. Alternatively, its involvement could be masked by compensation through redundant enzymes.”

*Line 588: What is the significant signal detected in noninfected cells (Figure 8A)?*

Thank you for making us aware of this. Uninfected cells were actually only employed as gating control for analysis of flow cytometry data. The “infected” signals in these samples are very few false positive events due to the gating, and presentation of these data is irrelevant for the experiment’s message. Thus, the figure has been corrected accordingly.

*Line 661: "Thus, a link between miltefosine sensitivity and 3'-nucleotidases/nucleases might be strain specific." I agree, but also point out that the resistance was linked to different members of this protein family located on chr 31.*

We thank for the helpful addition, it has has been added accordingly.

Lines 726-727: “Thus, a link between miltefosine sensitivity and 3’-nucleotidases/nucleases might be strain- or protein cluster-specific.”

Significance

*This study stands out for its use of primary human cells (macrophages and peripheral blood lymphocytes), the robustness of its statistical assessment, the breadth of complementary approaches applied, and the key findings that link p1/s1 to nutrient acquisition from hosts and to the subversion of host immune responses during early infection, specifically through the generation of free adenosine and the degradation of neutrophil extracellular traps (NETs). These findings provide important new insights that significantly advance our limited understanding of the role of parasite-secreted nucleotidases in infection and disease. As such, this study should be of interest to a broad audience with a focus on infection biology.*

Please define your field of expertise with a few keywords to help the authors contextualize your point of view:

*My expertise ranges from comparative genomics and molecular parasitology to the study of parasite immune evasion strategies, and is thus fully aligned with the focus of this manuscript.*
