# Supplementary material for: p1/s1, a 3’-nucleotidase/nuclease, allows Leishmania major to circumvent host innate immune response mechanisms
Source: PLoS Pathog. 2026 May 20;22(5):e1014197. doi: 10.1371/journal.ppat.1014197 (PMC13232948; doi:10.1371/journal.ppat.1014197)
Supplement: Attachment — Submitted filename: Response to the reviewers.docx [file ppat.1014197.s005.docx]

**Response to the reviewers**

We thank the editor and the reviewers for assessing our revised manuscript version and for the constructive and positive feedback.

In the following, we like to address the comments of Reviewer #1.

*Reviewer #1: Generally, they have also responded appropriately to the comments from the other reviewers. However, I think there are some additional clarifications required:

- For the additional wording in the methodology (lines 99-101) in which they explain the composition of the stationary phase culture can a reference to figure S1 be included for the apoptotic-like cells and a representative set of PNA data shown for the metacyclic rate.*

We appreciate the requested clarification of the stationary-phase parasites and adjusted the respective paragraph as follows:

Lines 99-102: “Logarithmic-phase (logPh) promastigotes, 3 days post passaging, or stationary-phase (statPh) promastigotes from 6-8 days post passaging were used for experiments. Stationary-phase promastigotes typically consist of approximately 2-10% metacyclic parasites and 50-60% dead parasites exhibiting apoptosis-like features, e.g. phosphatidylserine exposure (SupFig1).”

*Reviewer #1: - For the comment about the compensatory mechanism, I looked at the data presented in the thesis the authors linked to and it did not seem to include RNAseq data from the KO cell lines. It just had expression levels for the different genes in the p1/s1 array in log, stationary phase and axenic amastigotes. The mechanism for compensation is not the focus of this manuscript so in my opinion it is not necessary to look at but I just wanted to clarify which data they were alluding to in that thesis.*

*Reviewer #1: - In response to Reviewer 3’s comment about the 50% of stationary phase that are dead or undergoing cell death, the authors state the in their culture conditions they see 50-60% dead parasites but in the new section of the methods they are calling this apoptotic-like. Similarly, in the response to Comment 5 that focuses on Figure 4F, the authors state that the stationary phase cultures have a comparable proportion of dead parasites (+/- 5%). In this context are the dead parasites the 50-60% apoptotic-like cells?*

Yes. In this context the dead parasites exhibit apoptotic-like characteristics: rounding of the cell body, phosphatidylserine exposure and DNA fragmentation (data not shown).

We are confident that rephrasing of the paragraph in lines 99-102 (see above) now clarifies the composition and the appearance of the stationary-phase parasites.

*Reviewer #1:* Moreover, in the text (lines 371-381) the authors state that miltefosine induces 'early cell death processes' and stationary phase parasites 'undergo intrinsic cell death'. Can the authors clarify whether these are dead parasites or still alive but expressing apoptotic markers? There are a number of different phrases in the manuscript and the responses being used and I think it is important that there is consistency.

As described in lines 369-373, we employed low doses of drugs that would lead to cell death after 24 h. However, for the proteome analysis, we only treated the parasites for 3 h (miltefosine) or 6 h (staurosporine), meaning that intracellular apoptosis signaling is likely to be induced but parasites are still alive.

In lines 372-373, we added a short explanation to the existing sentence:

“Following treatment with miltefosine for 3 hours or staurosporine for 6 hours, with early cell death processes being induced (SupFig. 1) but not being lethal at these time points, we performed a quantitative proteome analysis to compare protein expression.”

The stationary phase parasites used for proteome analysis consisted of approximately 50% dead cells, as described before. This population harbors parasites that recently underwent intrinsic apoptosis or are in the process of doing so.

*Reviewer #1:* Finally, would the authors include an example gel to supplement Figure 5 which shows the endonuclease activity of the addback. They have it for the parentals and the KOs but not the addback. This could be included in a supplementary figure.

Many thanks for this comment. In the previous version of the manuscript, we accidentally missed including the gel data for the addback parasites in Fig 5E. A representative gel showing the endonuclease activity of parental, p1s1 knockout and addback parasites is now inserted into Fig 5E.
